# Inhibiting Receptor of Advanced Glycation End Products Attenuates Pressure Overload-Induced Cardiac Dysfunction by Preventing Excessive Autophagy

**DOI:** 10.3389/fphys.2018.01333

**Published:** 2018-09-24

**Authors:** Wenbin Gao, Zheng Zhou, Birong Liang, Yusheng Huang, Zhongqi Yang, Yang Chen, Lu Zhang, Cui Yan, Jiajia Wang, Lu Lu, Zhaorui Wen, Shaoxiang Xian, Lingjun Wang

**Affiliations:** ^1^The First Affiliated Hospital, Guangzhou University of Chinese Medicine, Guangzhou, China; ^2^Lingnan Medical Research Center, Guangzhou University of Chinese Medicine, Guangzhou, China; ^3^Guangzhou Key Laboratory of Chinese Medicine for Prevention and Treatment of Chronic Heart Failure, Guangzhou, China; ^4^School of Pharmaceutical Sciences, Guangzhou University of Chinese Medicine, Guangzhou, China

**Keywords:** receptor of advanced glycation end-products, autophagy, cardiac dysfunction, autophagic cell death, cardiac hypertrophy

## Abstract

The receptor for advanced glycation end products (RAGE) is involved in heart failure (HF) by mediating diverse pathologic processes, including the promotion of inflammation and autophagy. However, the role of RAGE in pressure overload-induced HF is not well understood. We found that stimulation of RAGE triggered the death of neonatal rat ventricular myocytes (NRVMs), while cell death was alleviated by ATG5 knockdown. Using transverse aortic constriction (TAC) in mice as a model of pressure overload-induced HF, we demonstrated that RAGE knockout or RAGE blockade attenuated cardiac hypertrophy and fibrosis as well as cardiac dysfunction at 8 weeks after TAC. Importantly, RAGE knockout reversed upregulation of autophagy related proteins (LC3BII/I and Beclin 1) and reduced cardiomyocyte death, indicating that excessive autophagy after TAC was inhibited. Moreover, RAGE knockout or blockade reduced the upregulation of pp65-NFκB and BNIP3, which mediate autophagy. Taken together, these results suggest that RAGE plays an important role in the progression of HF by regulating autophagy. Therefore, inhibition of the RAGE-autophagy axis could be a promising new strategy for treatment of heart failure.

## Introduction

Heart failure (HF) is a complicated clinical syndrome that represents the final stage of many cardiac disorders, and it results in multiple organ dysfunction and premature death. Although, treatments targeting neurohormonal signaling have somewhat improved the survival of patients with HF, it remains a major cause of morbidity and mortality worldwide. HF is classified into two major categories, which are heart failure with a reduced ejection fraction (HFrEF) and heart failure with a preserved ejection fraction (HFpEF). Both HFpEF and HFrEF share similar clinical features, but their etiology and treatment are different. In particular, HFpEF is usually associated with myocardial stiffness, whereas HFrEF is associated with impaired myocardial contractility and ventricular dilation (Borlaug and Redfield, 2011). Thus, understanding the mechanisms that lead to these different types of HF is essential for development of new therapies.

Pressure overload is a common cause of HF and cardiac remodeling. Stress on the heart promotes pathologic remodeling, with an increase of cardiomyocyte size, interstitial fibrosis, and inflammation, eventually leading to HF (van Berlo et al., 2013). Modulation of the signaling pathways that regulate these cellular processes could possibly prevent the progression of HF. A role of the advanced glycation end products (AGE)-receptor for AGE (RAGE) signaling pathway has recently been identified in the pathogenesis of diverse process, such as inflammation, malignancy, diabetes/diabetic complications, and neurodegeneration. In addition, the AGE-RAGE pathway has an essential role in heart disease. Accumulation of AGE is a major cause of asymptomatic diastolic and systolic cardiac dysfunction in patients with diabetes (Berg et al., 1999; Willemsen et al., 2011), and AGE may also be involved in the development and progression of HF in non-diabetic patients (Hartog et al., 2007). The level of circulating soluble RAGE was reported to be correlated with the severity of cardiac dysfunction (Raposeiras-Roubin et al., 2010; Wang et al., 2011). In addition, administration of a RAGE ligand (HMGB1) exacerbates ischemia/reperfusion injury, whereas RAGE-deficient mice show less myocardial damage after ischemia and reperfusion (Andrassy et al., 2008). Moreover, RAGE has been identified as a prognostic factor for patients with HF (Koyama et al., 2008). However, the mechanisms by which RAGE contributes to HF are not well understood.

Autophagy is an evolutionarily conserved process in which organelles and proteins undergo degradation, followed by recycling to maintain cellular homeostasis (Mizushima et al., 2008; Yang and Klionsky, 2010). There is evidence that basal autophagy plays a role in quality control and housekeeping in cardiomyocytes (Wang et al., 2008; Singh and Cuervo, 2011), while induction of autophagy by cardiac stress has a cytoprotective benefit (Yan et al., 2005; Nakai et al., 2007). However, excessive autophagic activity induced by pressure overload or reperfusion injury has been found to accentuate cardiac remodeling (Matsui et al., 2007; Zhu et al., 2007), indicating that autophagy can be detrimental in HF. Thus, modulation of autophagic activity could be a potential target for the treatment of patients with HF.

It has been reported that the AGE-RAGE pathway is involved in autophagic death of cardiomyocytes (Tsoporis et al., 2010; Hou et al., 2014), and that RAGE blockade improves cardiomyocyte viability through inhibiting the induction of autophagy by AGE (Hou et al., 2014). However, the role of RAGE-mediated autophagy in HF has not been investigated in detail. Therefore, we performed this study to explore the role of RAGE-mediated autophagy in HF induced by transverse aortic constriction (TAC), which leads to cardiac hypertrophy followed by ventricular dilatation and HFrEF over time. We examined the effect of inhibiting autophagy by RAGE blockade and demonstrated that it improved cardiac function after TAC. Importantly, we found that RAGE is a critical factor for regulation of autophagy in the heart, suggesting that treatment which targets RAGE-mediated autophagy could be promising for HF.

## Materials and Methods

### Cell Culture

Neonatal rat ventricular cardiomyocyte (NRVMs) were isolated from the left ventricles of Sprague-Dawley rats (1–3 days old). Briefly, ventricular tissues were digested with 1.0 mg/ml Type II Collagenase (Worthington Bio. Chem. Cat No. CLS-2). Then cells were incubated for 1 h in Dulbecco’s modified Eagle’s medium (DMEM) with 10% fetal bovine serum (FBS) to allow attachment of fibroblasts, after which the floating cardiomyocytes were cultured in DMEM supplemented with 0.1 M BrdU (Sigma, Cat No.B-5002), 10% FBS, and 1% penicillin-streptomycin (Thermo Fisher Scientific, Cat No.15140122). On day 4, BrdU was removed from the culture medium, and NRVMs were used for the experiments described below. NRVMs were treated with AGE (Millipore, Billerica, MA, United States Cat No.121800) at 25, 50, 100, 200, or 400 μg/ml for 48 h and cell viability was determined by using a Cell Counting Kit-8 (CCK-8, Dojindo, Cat No.CK04).

### Virus Production and Infection of NRVMs

NRVMs were infected with a recombinant lentivirus expressing rat RAGE or short hairpin RNA (shRNA) targeting rat RAGE (GeneChem, Shanghai, China) according to the manufacturer’s instructions. For infection with shRNAs targeting ATG5 and ATG7, the relevant sequences were cloned into the pLKO.1 vector. The lentivirus was packaged by co-transfection of pLKO.1-shATG5/7, psPAX, and pMD.2G using lipofectamine 2000. Virions were harvested at 48 and 72 h after transfection and used for infection.

### Animals

Male C57/BL mice weighing 18–22 g were purchased from the Laboratory Animal Center of Guangzhou University of Chinese Medicine. RAGE knockout mice (Myint et al., 2006) were a gift from Kanazawa University, Japan. The protocol for this study was approved by the Animal Care Committee of Guangzhou University of Chinese Medicine and was strictly in accord with the National Institutes of Health guidelines for Care and Use of Laboratory Animals.

### Mouse Model of Transverse Aortic Constriction

Surgery was performed to create TAC in mice according to the established protocol (Wagner et al., 2001). Briefly, wild-type mice and RAGE knockout mice were anesthetized by intraperitoneal injection of sodium pentobarbital (50 mg/kg, Sigma), and thoracotomy was done while the mice were connected to a ventilator for small animals. The aorta was ligated between the right innominate and left common carotid arteries using a 27G needle and an 8-0 suture. On postoperative day 1, the mice were randomly assigned to treatment with a RAGE antagonist (FPS-ZM1) at 1 mg/kg/day (TAC + FPS-ZM1 group, *n* = 10) or treatment with an autophagy inhibitor (3MA dissolved in PBS) at 10 mg/kg/day (TAC + 3MA group, *n* = 10). The sham group underwent the same thoracotomy procedure without aortic constriction. The wild-type TAC group and the RAGE knockout TAC group were administered 0.9% sodium chloride instead of the above drugs. Mice were sacrificed for analysis at 8 weeks postoperatively.

### Echocardiography

At 8 weeks after TAC or sham surgery, echocardiography was performed by using a Vevo 2,100 Imaging System (VisualSonics Inc., Toronto, ON, Canada) in mice under anesthesia with 1% isoflurane (RWD Life Science Co., Guangdong, China). The heart was examined in the short-axis view at the papillary muscle level and the average left ventricular (LV) internal dimension across at least 4 cardiac cycles was determined on M-mode images. Analysis of echocardiographic images was performed in a blinded manner.

### Histological Examination

Hearts were harvested and perfused with cold phosphate-buffered saline (PBS), followed by fixation in 4% paraformaldehyde overnight. Then the hearts were dehydrated and embedded in paraffin. Next, 5 μm thick sections were cut at the papillary muscle level for hematoxylin–eosin (H&E) and Masson’s trichrome staining. Each section was completely scanned by using a Caseviewer 2.0 (Panoramic 250/MIDI, 3D HISTECH, Hungary). For morphometric analysis, photographs were observed at 1× magnification to view the whole heart, and at 400× magnification to determine the single cardiomyocyte area. The relative fibrotic area was calculated by comparing the fibrotic area ratio (the ratio of the stained fibrotic area to the left ventricular area) in each treated group to that in the control group. Quantification of all data was done with Image J software.

### Immunohistochemistry and TUNEL Staining

Immunohistochemical staining was performed by using a Vectastain Elite ABC HRP Kit according to the manufacturer’s protocol. Briefly, sections were deparaffinized and rehydrated, incubated with blocking serum for 1 h, and then incubated with antibodies targeting RAGE (1:100, Abcam) or Beclin 1 (1:200, Abcam) for 60 min at room temperature. After being washed 3 times in PBS, the sections were incubated with the biotinylated secondary antibody for 30 min, and detection was performed with ABC reagent. Cell death was detected by using paraffin sections and the TMR Red *In situ* Death Detection Kit (Roche, Indianapolis, IN, United States) according to the manufacturer’s instructions. Nuclei were counterstained with DAPI.

### Quantitative Real-Time PCR

Total RNA was extracted from LV tissue by using an RNAprep Pure Tissue Kit (Tiangen, Beijing, China), after which first-strand cDNA was synthesized with a FastKing RT Kit (Tiangen, Beijing, China) and quantitative PCR (qPCR) was performed using SYBR^®^ Premix Ex Taq^TM^ II (Takara, Beijing, China). Target gene expression was normalized for that of GAPDH and compared among the groups. Details of the primers are provided in **Table 1**.

**Table 1:** Primers for Q-RT-PCR.

ANP/Mouse	Forward	AGGCAGATCATCAAGCCAGT
	Reverse	ACACACCACAAGGGCTTAGG
BNP/Mouse	Forward	AACGGTGAGCACCTACCTTG
	Reverse	TGACAGGCTGCAGGAGTATG
Fibronectin/Mouse	Forward	TGCAGTGACCAACATTGATCGC
	Reverse	AAAAGCTCCCGGATTCCATCC
CTGF/Mouse	Forward	ACTATGATGCGAGCCAACTGC
	Reverse	TGTCCGGATGCACTTTTTGC
Type 1 Collagen/Mouse	Forward	TGGCCTTGGAGGAAACTTTG
	Reverse	CTTGGAAACCTTGTGGACCAG
GAPDH/Mouse	Forward	GGCTGTATTCCCCTCCATCG
	Reverse	CCAGTTGGTAACAATGCCATGT
Atg5/Rat	Forward	TGGACCATCAACCGGAAACT
	Reverse	CAAGGGTATGCAGCTGTCCA
Atg7/Rat	Forward	GAGAGCCGATGGCTTCCTAC
	Reverse	CAGGTCAGCAGGTGCTACAA
GAPDH/Rat	Forward	GACATGCCGCCTGGAGAAAC
	Reverse	AGCCCAGGATGCCCTTTAGT
RAGE/genotype	Primer 1	CCAGAGTGACAACAGAGCAGAC
	Primer 2	CCTCGCCTGTTAGTTGCCCGAC
	Primer 3	GGTCAGAACATCACAGCCCGGA


### Western Blotting

LV tissues were lysed in a lysis buffer containing RIPA Lysis Buffer (Ca. P0013B, Beyotime, Shanghai, China), 1 mM PMSF (Ca. 78830, Sigma), and 1X Protease/Phosphatase Inhibitor Cocktail (Ca. 5872, CST). Then the lysates were subjected to SDS-PAGE (10 and 12%) and transferred to 0.45 μm PVDF membranes (Ca. IPVH00010, EMD Millipore, Billerica, MA, United States). The membranes were blocked with 5% skim milk (Ca. 9999, CST) at room temperature and then were incubated overnight at 4°C with the primary antibody directed against RAGE (ab3611, 1:1000; ab172473, 1:1000), LC3B (ab48394, 1:1000), Beclin 1 (ab62557, 1:2000), p65-NFκB (CST 8242, 1:1000), p-p65NFκB (CST 3033, 1:1000), BNIP3 (ab10433, 1:2000), or β-actin (CST 3700, 1:2000). After incubation with the secondary antibody for 2 h at room temperature, the blots were visualized by using Immobilon Western Chemiluminescent HRP Substrate (Ca. WBKLS0500, Millipore, Bedford, MA, United States).

### Statistical Analysis

Results are expressed as the mean ± SEM. Analyses were performed by two-way ANOVA, followed by the multiple comparisons test with a *post hoc* Student–Newman–Keuls test. SPSS 16.0 software was employed for all analyses and *P* < 0.05 was considered to indicate statistical significance.

## Results

### The AGE-RAGE Pathway Triggers Autophagic Death of Cardiomyocytes

**FIGURE 1:**
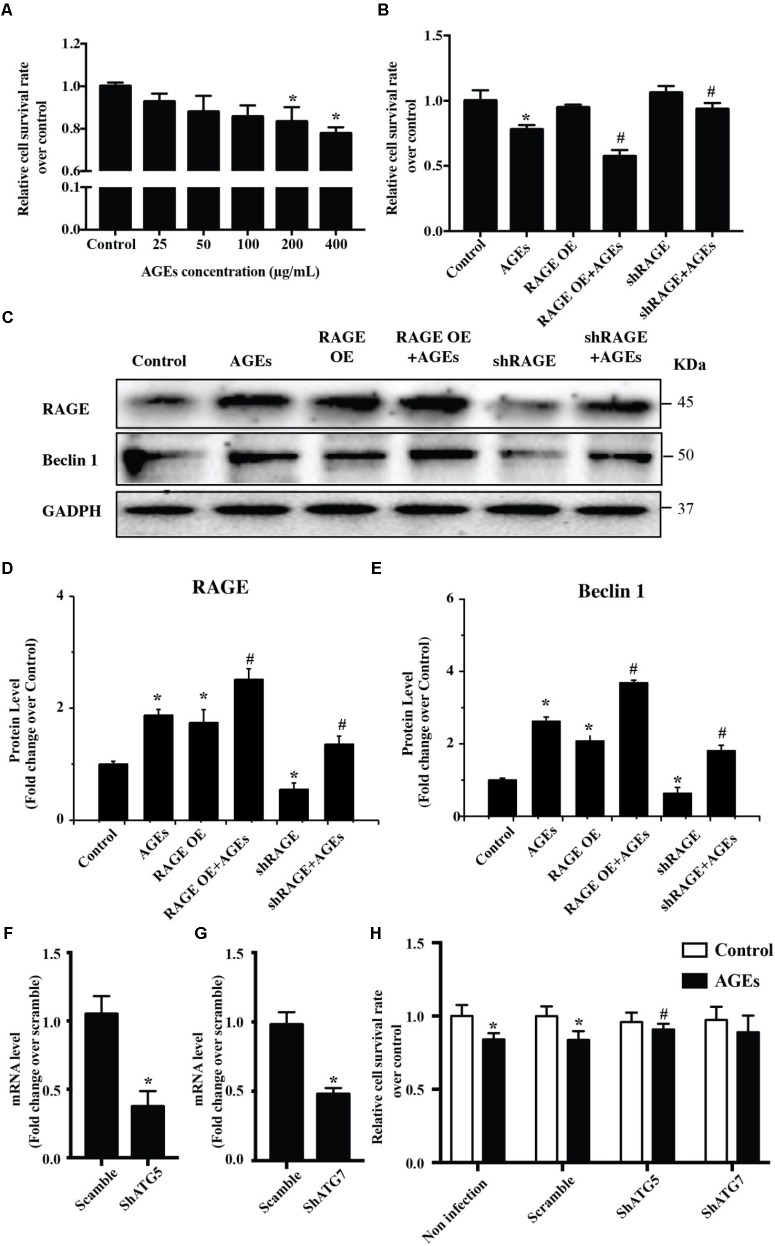
The AGE-RAGE pathway triggers autophagic death of cardiomyocytes. **(A)** Viability of NRVMs was assessed by the CCK8 assay after treatment with various concentrations of AGE. **(B)** Cell viability measured by the CCK8 assay after AGE treatment (400 μg/ml) in NRVMs with RAGE overexpression or knockdown. **(C)** Expression of RAGE and Beclin 1 were detected by western blotting after AGE treatment of NRVMs with RAGE overexpression or knockdown. Representative figures are from different blots in **Supplementary Figure S1**. **(D,E)** Quantitative analysis of RAGE and Beclin 1 protein levels. **(F,G)** NRVMs were infected with short hairpin RNA (shRNA) targeting ATG5, ATG7, or the scramble control. Expression of ATG5 and ATG7 was detected by q-RT-PCR. **(H)** Knockdown of ATG5 reduced the AGE-induced death of NRVMs. *n* = 3. Data are the mean ± SE., ^∗^*P* < 0.05 vs. control, ^#^*P* < 0.05 vs. AGE-treated scramble group.

To study the potential influence of AGE-RAGE on cardiomyocyte growth, we examined the viability of NRVMs after AGE treatment by using the CCK8 assay. This assay showed that AGE reduced NRVMs viability in a dose-dependent manner (**Figure 1A**). To test whether AGE influenced NRVM viability via RAGE, we assessed the viability of AGE-treated NRVMs with RAGE overexpression or knockdown. Overexpression of RAGE led to a slight increase of cell death in the absence of AGE treatment. In addition, RAGE-overexpressing cells showed a marked increase of death after AGE treatment compared to wild-type NRVMs, whereas cells with RAGE knockdown showed reduction of death after AGE treatment (**Figure 1B**). Western blotting with a RAGE antibody revealed that AGE treatment increased RAGE expression by NRVMs and that RAGE expression was further elevated by AGE treatment of RAGE-overexpressing cells. On the other hand, the response to AGE treatment was decreased in cells with RAGE knockdown (**Figures 1C–E** and **Supplementary Figure S1**). Importantly, we observed that Beclin 1 (a protein related to autophagy) was increased after AGE treatment, a finding consistent with a previous report that AGE can trigger autophagy (Hou et al., 2014). Furthermore, Beclin 1 expression was further elevated by AGE treatment of RAGE-overexpressing NRVMs, while the response of Beclin 1 to AGE treatment was reduced in NRVMs with RAGE knockdown. We then hypothesized that the AGE-RAGE pathway could trigger autophagic death of NRVMs. To test this hypothesis, we studied the effect of ATG5/ATG7 knockdown on NRVM viability after AGE treatment. While ATG5/ATG7 knockdown did not cause either death or proliferation of NRVMs in the absence of AGE treatment, ATG5/ATG7 knockdown led to reduction of cell death after AGE treatment (**Figures 1F–H**). Taken together, our results suggested that the AGE-RAGE pathway triggered autophagic death of cardiomyocytes.

### RAGE Knockout or Blockade Attenuates Cardiac Dysfunction at 8 Weeks After TAC

**FIGURE 2:**
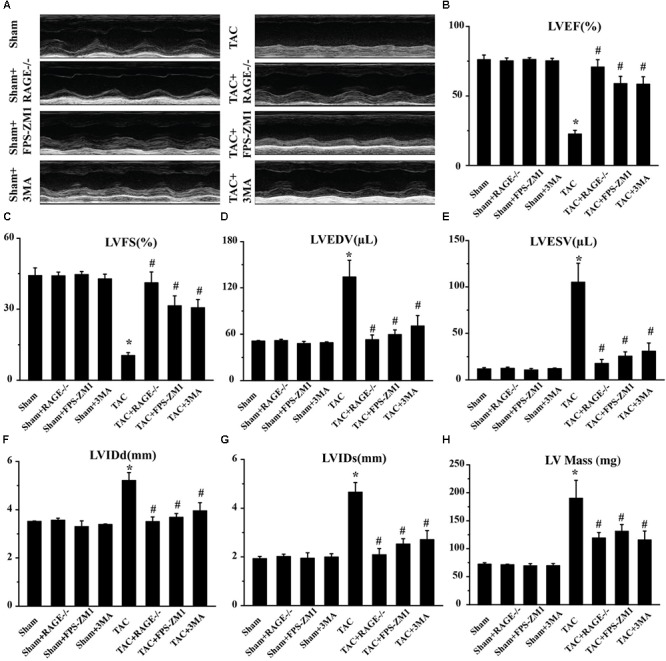
Amelioration of cardiac dysfunction at 8 weeks after TAC by genetic deletion of RAGE, a specific RAGE inhibitor (FPS-ZM1), or an autophagy inhibitor (3MA). **(A)** Representative M-mode images. **(B–H)** Echocardiography was performed to measure LVEF **(B)**, LVFS **(C)**, LVEDV **(D)**, LVESV **(E)**, LVIDd **(F)**, LVIDs **(G)**, and LV Mass **(H)**. Sham, sham + RAGE-/-, sham + FPS-ZM1, sham + 3MA, *n* = 10; TAC, TAC + RAGE-/-, TAC + FPS-ZM1, TAC + 3MA, *n* = 10. Data are the mean ± SE., ^∗^*P* < 0.05 vs. sham group^#^*P* < 0.05 vs. TAC group.

We then hypothesized that RAGE might play an important role in HF, since AGE-RAGE induced autophagic cell death and cardiomyocyte death are essential for progression of HF (van Empel et al., 2005). To test this hypothesis, we examined cardiac function in mice with RAGE knockout (Myint et al., 2006; **Supplementary Figure S2**) and mice with RAGE blockade mice after TAC. Compared to sham mice, echocardiography showed significant reduction of the left ventricular ejection fraction (LVEF) and left ventricular fractional shortening (LVFS) in TAC mice, as well as an increase of left ventricular end-diastolic volume (LVEDV), left ventricular end-systolic volume (LVESV), the end-diastolic left ventricular internal dimension (LVIDd), the end-systolic left ventricular internal dimension (LVIDs), and left ventricular mass (LV mass) (**Figure 2** and **Supplementary Figure S3**). In contrast, mice with RAGE knockout (TAC + RAGE-/- group) or RAGE blockade (TAC + FPS-ZM1 group) showed a significant increase of LVEF and LVFS, as well as a significant decrease of LVEDV, LVESV, LVIDd, LVIDs, and LV mass, when compared to control TAC mice with HF assessed at 8 weeks. These results indicated that RAGE knockout and RAGE blockade could improve cardiac dysfunction.

### RAGE Knockout or Blockade Suppresses Cardiac Hypertrophy in TAC Mice

**FIGURE 3:**
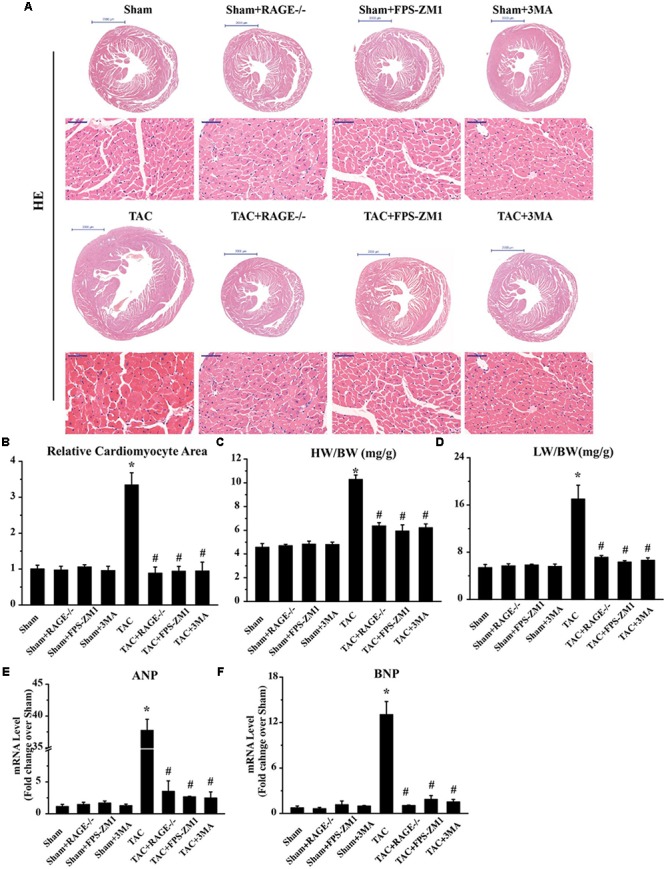
Suppression of cardiac hypertrophy after TAC by inhibition of RAGE and autophagy. **(A)** Representative HE-stained transverse heart sections and left ventricular sections are shown (scale bar: transverse sections = 2,000 μm, left ventricular sections = 50 μm). **(B)** The cardiomyocyte cross-sectional area was measured with a quantitative digital image analysis system. **(C)** Heart/body weight ratio (HW/BW), and **(D)** lung/body weight ratio (LW/BW). Cardiac tissue expression of mRNA for genes associated with hypertrophy, ANP **(E)**, and BNP **(F)**. GAPDH was used as the internal control. *n* = 5. Data are the mean ± SE, ^∗^*P* < 0.05 vs. sham group ^#^*P* < 0.05 vs. TAC group.

To investigate whether RAGE knockout or blockade could ameliorate cardiac hypertrophy after TAC, we analyzed cardiomyocyte size by examining H&E-stained cardiac sections. We also calculated the heart weight/body weight ratio and the lung weight/body weight ratio, as well as determining the expression of hypertrophy marker gene mRNAs by qPCR. Histological examination of cardiac sections showed that TAC mice had a larger total heart size and larger single cardiomyocyte area compared to sham mice (**Figures 3A,B**). TAC mice also had a significantly larger heart weight/body weight ratio (HW/BW, **Figure 3C**) and lung weight/body weight ratio (LW/BW, **Figure 3D**) than sham mice. Furthermore, expression of the pro-hypertrophic genes ANP (**Figure 3E**) and BNP (**Figure 3F**) was upregulated in TAC mice, confirming that TAC caused cardiac hypertrophy. Both RAGE knockout and RAGE blockade significantly reduced the heart size and the single cardiomyocyte area after TAC. Consistent with these findings, the HW/BW, LW/BW, and expression of ANP and BNP were all significantly decreased by RAGE knockout or RAGE blockade. These findings indicated that RAGE knockout or blockade could prevent induction of cardiac hypertrophy by pressure overload.

### RAGE Knockout or Blockade Suppresses Cardiac Fibrosis in TAC Mice

**FIGURE 4:**
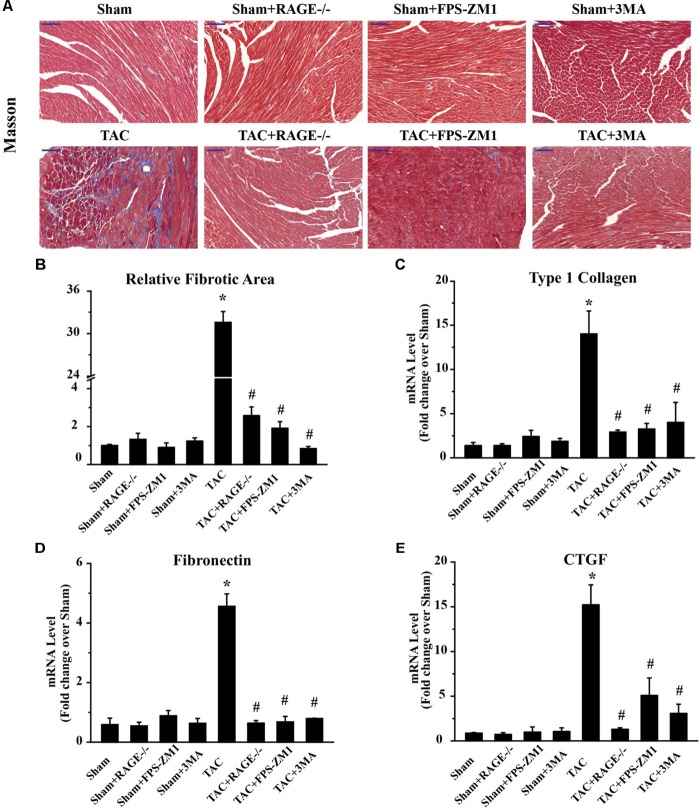
Effect of inhibiting RAGE or autophagy on cardiac fibrosis after TAC. **(A)** Myocardial fibrosis was detected by Masson’s trichrome staining (scale bar = 100 μm). Blue areas indicate fibrosis. **(B)** The fibrosis area was measured with a quantitative digital image analysis system, and mRNAs for fibrosis-associated genes [type 1 collagen **(C)**, fibronectin **(D)**, and CTGF **(E)**] were measured by q-RT-PCR. GAPDH was used as the internal control. *n* = 5. Data are the mean ± SE., ^∗^*P* < 0.05 vs. sham, group ^#^*P* < 0.05 vs. TAC group.

To investigate whether RAGE knockout or blockade suppressed the development of cardiac fibrosis in response to pressure overload, we analyzed the fibrosis area by histological examination of sections with Masson’s trichrome staining and we also assessed the expression of fibrosis-related genes by qPCR. The fibrosis area was significantly increased in TAC mice compared with sham mice. In contrast, there was significant reduction of the fibrosis area in both RAGE knockout mice and RAGE blockade mice compared with TAC mice (**Figures 4A,B**). In agreement with these results, expression of pro-fibrotic genes [type I collagen, fibronectin, and connective tissue growth factor (CTGF)] showed marked upregulation in TAC mice, while upregulation of these genes was reduced in RAGE knockout or RAGE blockade mice (**Figures 4C–E**). These results demonstrated that RAGE was essential for progression of cardiac fibrosis.

### RAGE Knockout or Blockade Inhibits Excessive Autophagy After TAC

**FIGURE 5:**
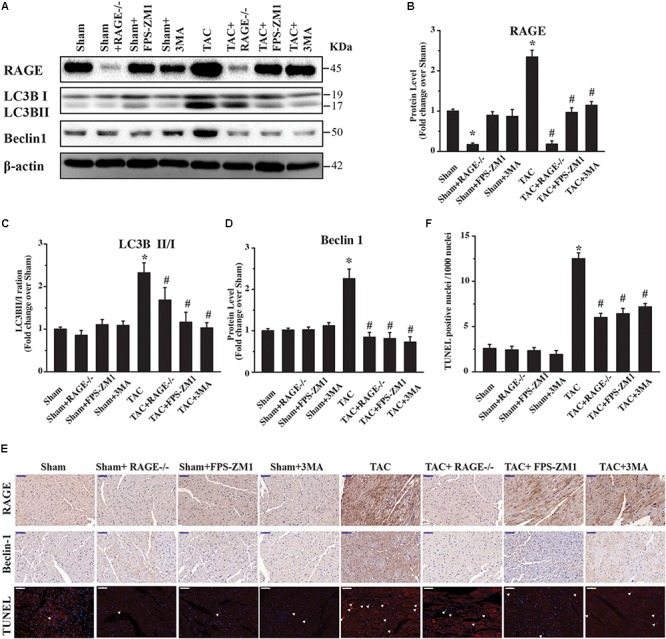
Excessive RAGE-mediated autophagy leads to cardiac dysfunction. Left ventricular levels of RAGE and autophagy-related proteins were assessed by western blotting after 8 weeks of pressure overload. **(A)** Pressure overload upregulated RAGE expression. It also induced conversion of LC3B-I to LC3B-II and activation of Beclin 1, which are two markers of autophagy. Genetic deletion or specific inhibition of RAGE prevented excessive autophagy by reducing conversion of LC3B-I to LC3B-II and suppressing Beclin 1, while the autophagy inhibitor 3MA had similar effects. Representative figures are from different blots in **Supplementary Figure S4**. **(B–D)** Quantification of RAGE, LC3-II/I, and Beclin 1. **(E)** Immunohistochemical staining of cardiac sections for RAGE or Beclin 1, and TUNEL staining at 8 weeks after TAC (scale bar = 50 μm). Arrowheads indicate TUNEL-positive nuclei. For western blot, *n* = 4; for immnuo staining and TUNEL, *n* = 5. **(F)** Quantification of TUNEL positive nucleis. For the TUNEL assay, 4 areas of each heart section were examined. Data are the mean ± SE., ^∗^*P* < 0.05 vs. sham group ^#^*P* < 0.05 vs. TAC group.

Because RAGE induced autophagic cell death *in vitro* (**Figure 1**), we hypothesized that RAGE promoted cardiac dysfunction and hypertrophy in HF by inducing excessive autophagy. To investigate this idea, we first analyzed the expression of RAGE and other proteins related to autophagy in mice. Western blotting showed that RAGE expression was upregulated in TAC mice (**Figures 5A,B** and **Supplementary Figure S4**), along with a significant increase in the expression of proteins related to autophagy, LC3BII/I and Beclin 1 (**Figures 5A,C,D**). Notably, the LC3BII/I ratio and Beclin 1 protein expression were significantly decreased in RAGE knockout mice and RAGE blockade mice compared with TAC mice (**Figures 5A,C,D**). In agreement with this finding, immunostaining showed that upregulation of RAGE and Beclin 1 expression in the heart after TAC was reversed by inhibition of RAGE (**Figure 5E**). To investigate whether inhibition of RAGE attenuated cardiac dysfunction after TAC by reducing cell death *in vivo*, we performed the terminal deoxynucleotidyl transferase dUTP nick end labeling (TUNEL) assay. We found that TAC significantly increased cell death (**Figures 5E,F**), while cell death after TAC was significantly reduced in mice with RAGE knockout or RAGE blockade (**Figures 5E,F**). Moreover, inhibition of autophagy by 3-MA improved cardiac dysfunction and remodeling after TAC (**Figures 2–4**), in association with reduction of the LC3BII/I ratio and Beclin 1 expression as well as a decrease of cell death (**Figures 5A–F**). These results suggested that RAGE knockout or blockade improved cardiac function by reducing cell death through suppression of excessive autophagy after TAC.

### RAGE Regulates Autophagy Through p65-NFκB and BNIP3

**FIGURE 6:**
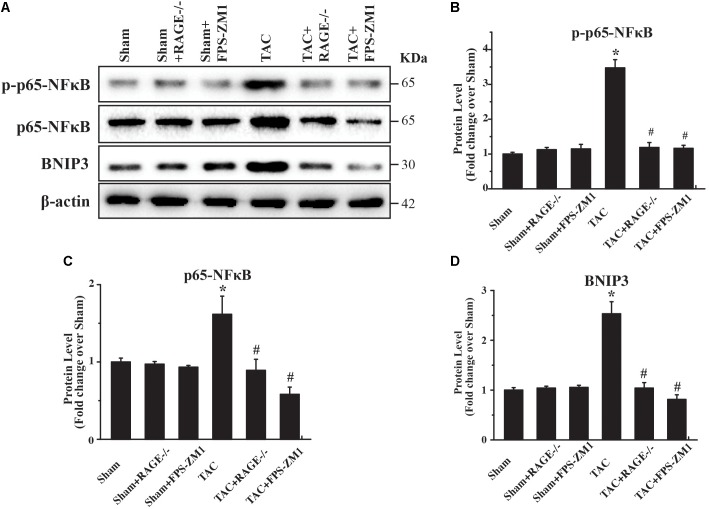
RAGE upregulates autophagy via the NFκB-BNIP3 pathway. **(A)** Western blotting reveals activation of NFκB and BNIP3 in the left ventricular myocardium at 8 weeks after TAC. Inhibition of RAGE suppressed expression of phosphorylated and total p65-NFκB and expression of BNIP3, while an autophagy inhibitor had similar effects. Representative figures are from different blots in **Supplementary Figure S5** **(B–D)** Quantitative analysis of phosphorylated p65-NFκB, total p65-NFκB, and BNIP3 protein levels. *n* = 4. Data are the mean ± SE., ^∗^*P* < 0.05 vs. sham group, ^#^*P* < 0.05 vs. TAC group.

To investigate the mechanism underlying RAGE-mediated autophagy in HF, we examined the expression of several regulators of autophagy (p65-NFκB, phophos-p65-NFκB (pp65-NFκB), and BNIP3) by western blotting. We found significant upregulation of both p65-NFκB and pp65-NFκB expression in TAC mice compared with sham mice (**Figures 6A–C** and **Supplementary Figure S5**). Importantly, RAGE knockout and RAGE blockade both prevented the increase of p65-NFκB and pp65-NFκB expression after TAC. Because p65-NFκB activates the autophagy related gene Beclin 1 (Copetti et al., 2009), it is likely that deletion of RAGE suppressed autophagic activity through downregulation of p65-NFκB. Interestingly, RAGE knockout or blockade also prevented an increase in expression of the autophagy regulator BNIP3 in TAC mice (**Figures 6A,D**). Considering that NFκB regulates BNIP3, our findings suggested that RAGE knockout or RAGE blockade suppress excessive autophagy in this mouse model of HF possibly by modulating the NFκB/BNIP3/Beclin 1 pathway.

## Discussion

RAGE plays an essential role in various processes causing cell damage, such as oxidative stress and inflammation (Ray et al., 2016), and RAGE blockade has been shown to have a beneficial effect on heart disease (Bangert et al., 2016). In the present study, we demonstrated that RAGE blockade ameliorated TAC-induced cardiac dysfunction by inhibiting excessive autophagy. In addition, RAGE knockout inhibited the development of cardiac hypertrophy and cardiac fibrosis in TAC mice, as well as enhancing cardiac function. We also showed that RAGE is a critical regulator of autophagy in HF, since RAGE knockout reduced the expression of BNIP3 and p65-NFκB to prevent overexpression of proteins related to autophagy (LC3BII/I and Beclin 1) after TAC. Thus, RAGE-mediated autophagy regulated the progression of HF in the present TAC model and RAGE could be a potential target for treating HF induced by pressure overload.

It has been unclear whether RAGE directly regulates excessive autophagy or autophagy occurs as a consequence of TAC-induced HF. The present study provided evidence that RAGE induces cardiac autophagy during HF. We demonstrated that treatment with AGE or overexpression of RAGE induced the expression of Beclin 1 (a gene related to autophagy) in NRVMs, while RAGE knockout reduced Beclin 1 expression. Moreover, RAGE knockout or blockade significantly decreased the myocardial expression of autophagy related proteins (LC3BII/I and Beclin 1) after TAC, suggesting that autophagic activity was reduced in mice with knockout or blockade of RAGE. Consistent with this finding, previous studies have shown that RAGE mediates autophagy in other cellular process (Kang et al., 2011a,b; Hou et al., 2014). Therefore, our results suggest that RAGE may directly regulate autophagic activity both *in vitro* and *in vivo*.

An important finding of this study was that activation of RAGE may induce excessive autophagy, leading to autophagic cell death and eventually contributing to progression of HF. Previous study shows that treatment of FPS-ZM1 attenuated cardiac remodeling after TAC via enhancing phosphorylation of AMPK and reducing phosphorylation of mTOR (Liu et al., 2016). In our study, we further show that deletion of RAGE could attenuate the HF progression, which could be more directly characterizing the role of RAGE after TAC. Importantly, our study has identified a new mechanism that targeting RAGE mediated autophagy exerts the benifical effect after TAC. We showed that exposure of NRVMs to AGE caused a dose-dependent increase of cell death, while this could be prevented by RAGE knockout. Consistently, expression of the autophagy related protein Beclin 1 was decreased by RAGE knockout, suggesting that the AGE-RAGE pathway could trigger autophagic death of cultured NRVMs. Because RAGE is known to promote autophagic activity *in vivo*, it seems likely that RAGE induces excessive autophagy that leads to cardiomyocyte death during progression of HF.

Autophagy can have either a protective or detrimental effect on the heart. For example, excessive autophagic activity in response to pressure overload can be detrimental (Zhu et al., 2007). On the other hand, a recent study showed that mitochondrial autophagy is stimulated after downregulation of general autophagy and can ameliorate pressure overload-induced cardiac dysfunction (Shirakabe et al., 2016). These contradictory observations suggest that target-specific autophagy has an important role in cardiomyocyte health, while excessive accumulation of autophagosomes may impair cardiomyocyte function. Consistent with this idea, cardiotoxicity of doxorubicin was reported to be due to accumulation of autophagosomes (Li et al., 2016). Therefore, it is critical to consider these discrepancies when targeting autophagy for the treatment of HF. In this study, we showed that RAGE knockout or blockade significantly reduced LV myocardial expression of two potential regulators of autophagy (BNIP3 and p65-NFκB) in TAC mice at 8 weeks after surgery. This decrease of pp65-NFκB/BNIP3 expression following RAGE knockout suggests a cardioprotective effect. Another study has shown a cardioprotective role of NFκB (Misra et al., 2003), which has been reported to suppress BNIP3 expression under pro-apoptotic conditions (Shaw et al., 2008). These differing findings suggest that different disease mechanisms may alter distinct pathways related to NFκB, which is involved in regulation of various molecular pathways, including those for anti-apoptotic and autophagic processes (Baldwin, 2012). It is possible that RAGE mediates an NFκB signaling pathway involved in continuous activation of autophagy in mice with TAC-induced HF. It was previously demonstrated that autophagy is elevated for 3 weeks after induction of pressure overload and that this excessive autophagic response contributes to HF (Zhu et al., 2007). Accordingly, our results suggest that RAGE-NFκB/BNIP3/Beclin 1 may be one of the major contributors to excessive autophagy associated with TAC-induced HF.

## Conclusion

Our findings demonstrated that RAGE plays a key role in the progression of HF in mice with TAC, and RAGE knockout or RAGE blockade can prevent cardiac remodeling. Importantly, we found that RAGE knockout reduced excessive autophagic activity, suggesting the RAGE autophagy axis could be an attractive target for treatment of HF.

## Data Availability

The supporting data and materials presented in this publication are available upon request.

## Author Contributions

WG was responsible for the *in vitro* and *in vivo* experiments as well as data interpretation and manuscript writing. ZZ, BL, YH, LZ, and CY was responsible for generation of the animal model and performed the histological data analysis. JW, LL, and ZW carried out the cell cultures. ZY and YC were responsible for conception and design of the experiments. LW and SX was responsible for the conception and design of the study, edited the manuscript, and provided the funding. All authors read and approved the manuscript for publication.

## Conflict of Interest Statement

The authors declare that the research was conducted in the absence of any commercial or financial relationships that could be construed as a potential conflict of interest. The reviewer CR and handling Editor declared their shared affiliation.

## Supplementary Material

FIGURE S1 Molecular weight markers to gel data and scans in **Figure 1C**.

FIGURE S2 **(a)** PCR Genotyping of RAGE knockout mice. Bands of 380 and 301 bp are derived from the wild-type and knockout alleles, respectively. +/+, wild-type; -/-, homozygous RAGE knockout. **(b)** Western blot of WT and RAGE KO mice lung tissues using RAGE antibody.

FIGURE S3 Short axis B-mode of representative echo figure. Scale bars for time and dimensions are listed in lower panel.

FIGURE S4 Molecular weight markers to gel data and scans in **Figure 5A**.

FIGURE S5 Molecular weight markers to gel data and scans in **Figure 6A**.

## Abbreviations

AGE: advanced glycation end products
ANP: atrial natriuretic peptide
BNP: brain natriuretic peptide
HW/BW: heart to body weight ratio
LVEDV: left ventricular end diastolic volume
LVEF: left ventricular ejection fraction
LVESV: left ventricular end systolic volume
LVFS: left ventricular fractional shortening
LVIDd: end diastolic left ventricular internal dimension
LVIDs: end systolic left ventricular internal dimension
LV Mass: left ventricular mass
LW/BW: lung to body weight ratio
RAGE: receptor for advanced glycation end products
