# Supplementary figures and images for: Inhibiting Receptor of Advanced Glycation End Products Attenuates Pressure Overload-Induced Cardiac Dysfunction by Preventing Excessive Autophagy
Source: Front Physiol. 2018 Sep 24;9:1333. doi: 10.3389/fphys.2018.01333 (PMC6165873; doi:10.3389/fphys.2018.01333)

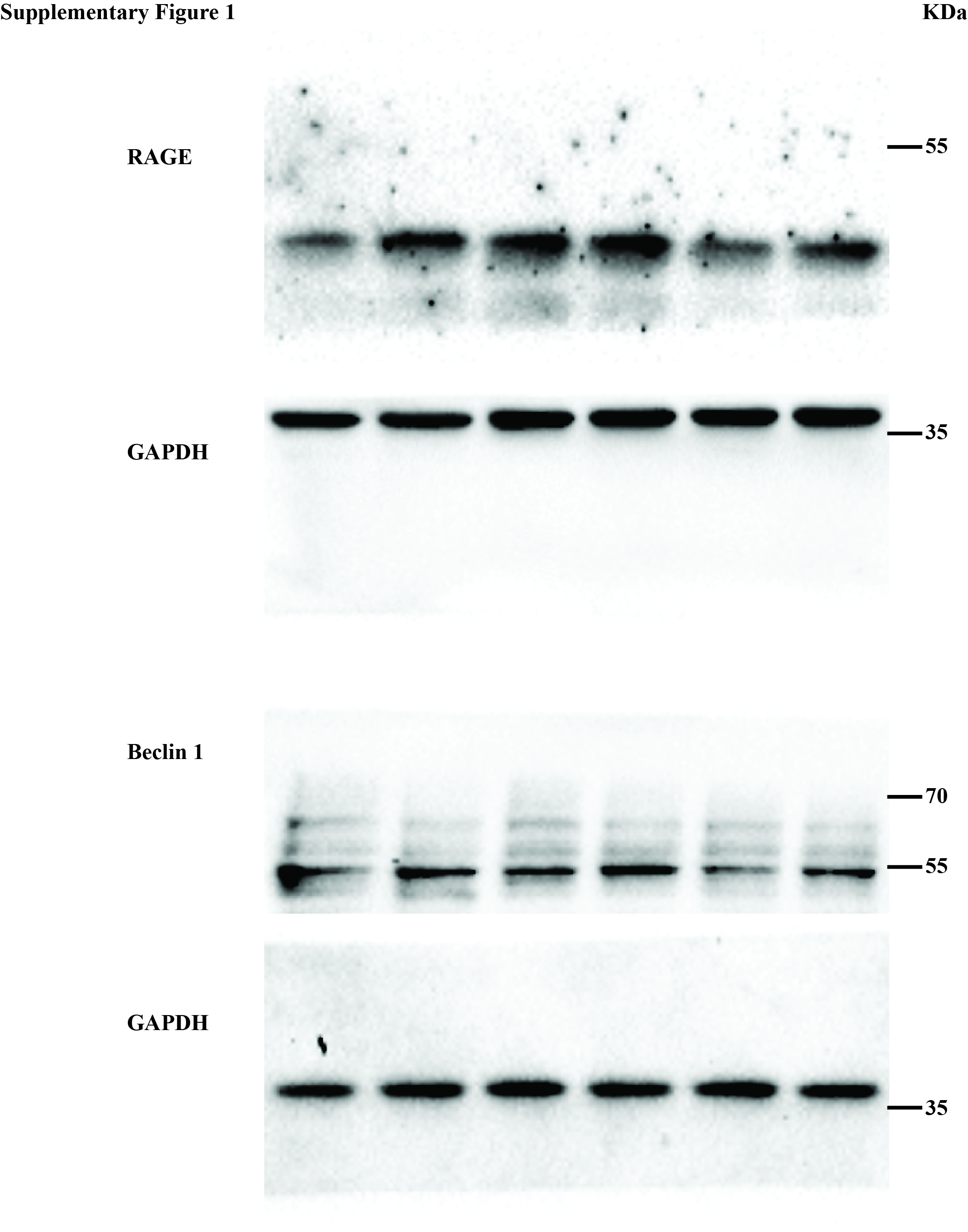

Supplement: FIGURE S1 — Molecular weight markers to gel data and scans in Figure 1C. [file Image_1.JPEG]

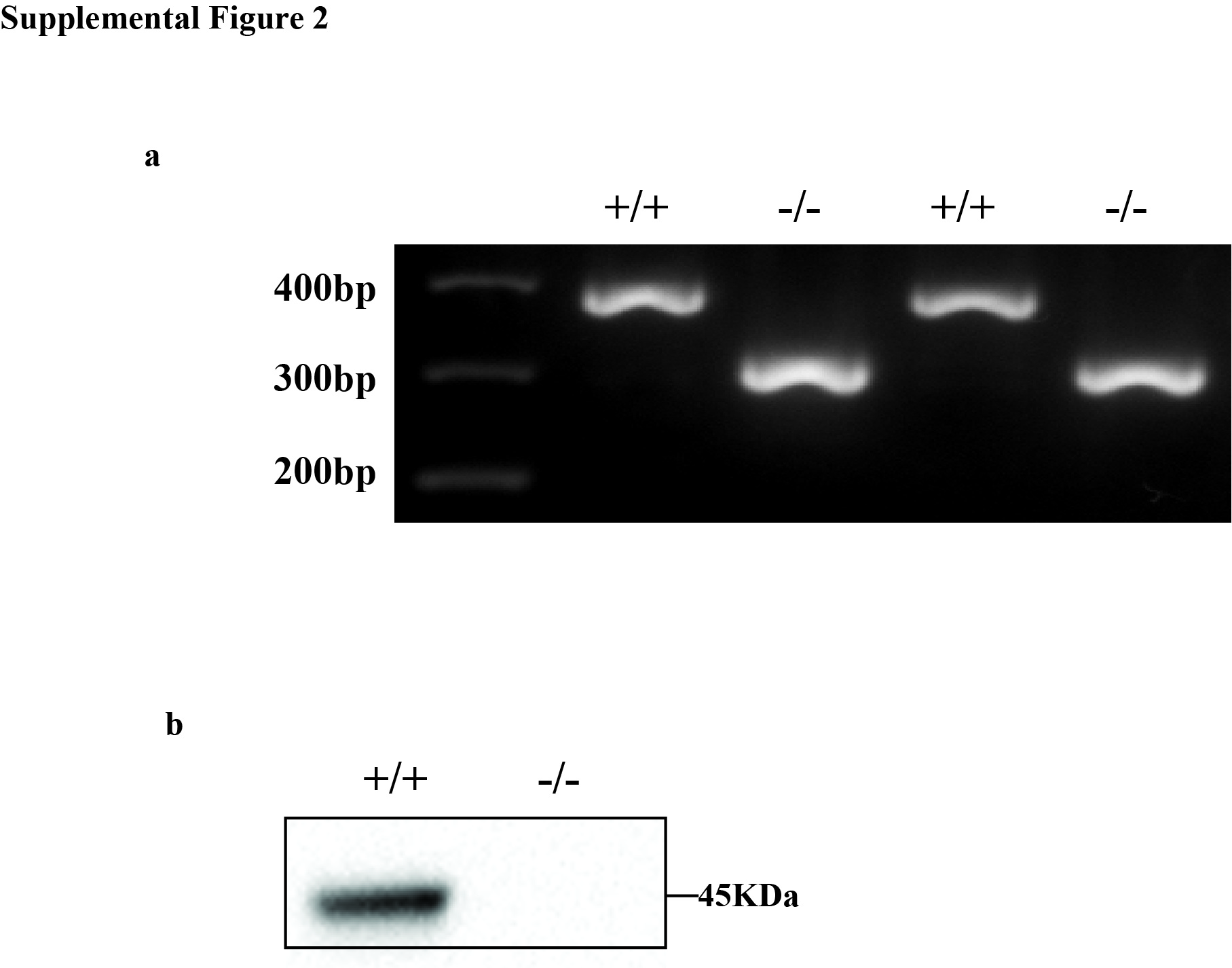

Supplement: FIGURE S2 — (a) PCR Genotyping of RAGE knockout mice. Bands of 380 and 301 bp are derived from the wild-type and knockout alleles, respectively. +/+, wild-type; -/-, homozygous RAGE knockout. (b) Western blot of WT and RAGE KO mice lung tissues using RAGE antibody. [file Image_2.JPEG]

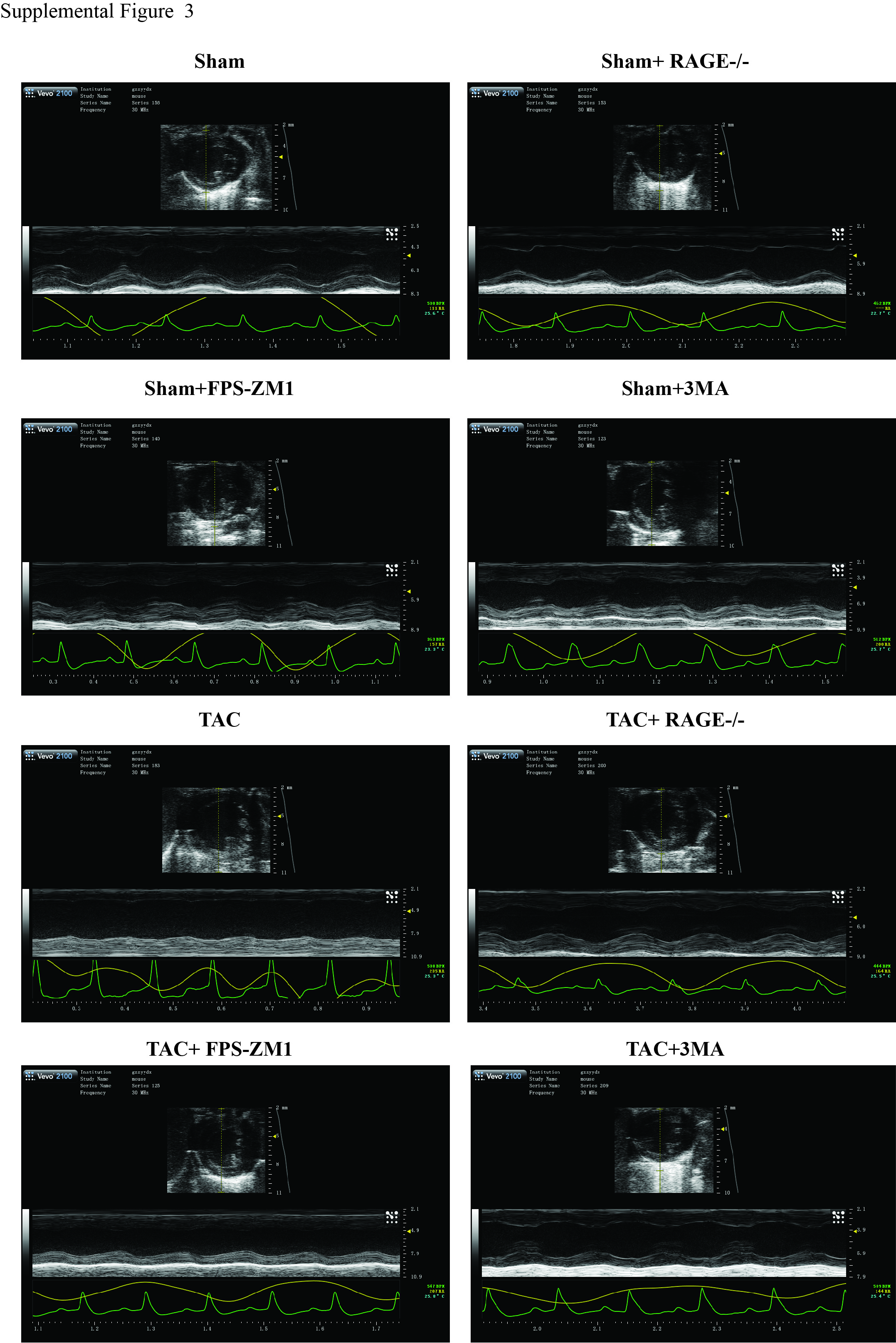

Supplement: FIGURE S3 — Short axis B-mode of representative echo figure. Scale bars for time and dimensions are listed in lower panel. [file Image_3.JPEG]

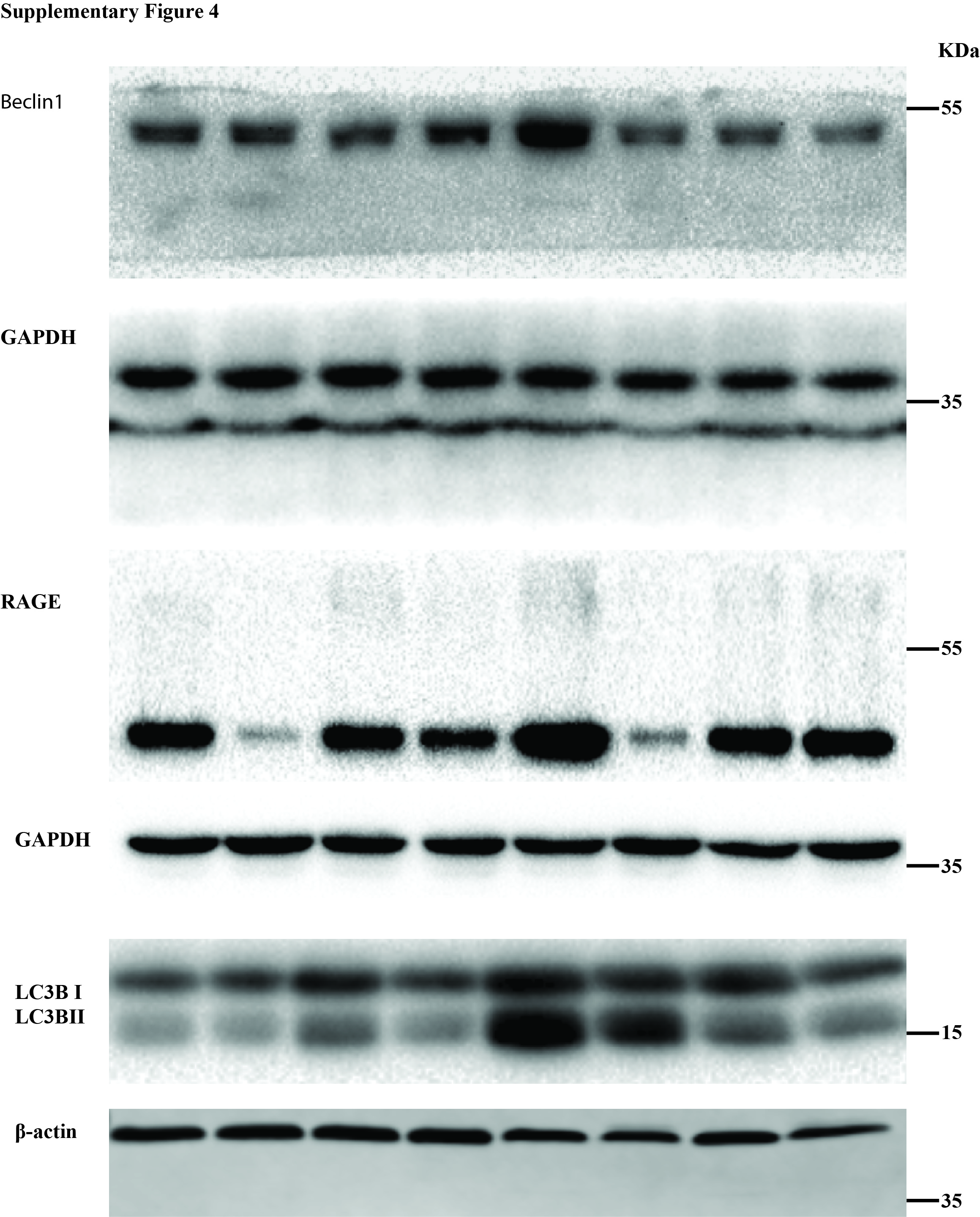

Supplement: FIGURE S4 — Molecular weight markers to gel data and scans in Figure 5A. [file Image_4.JPEG]

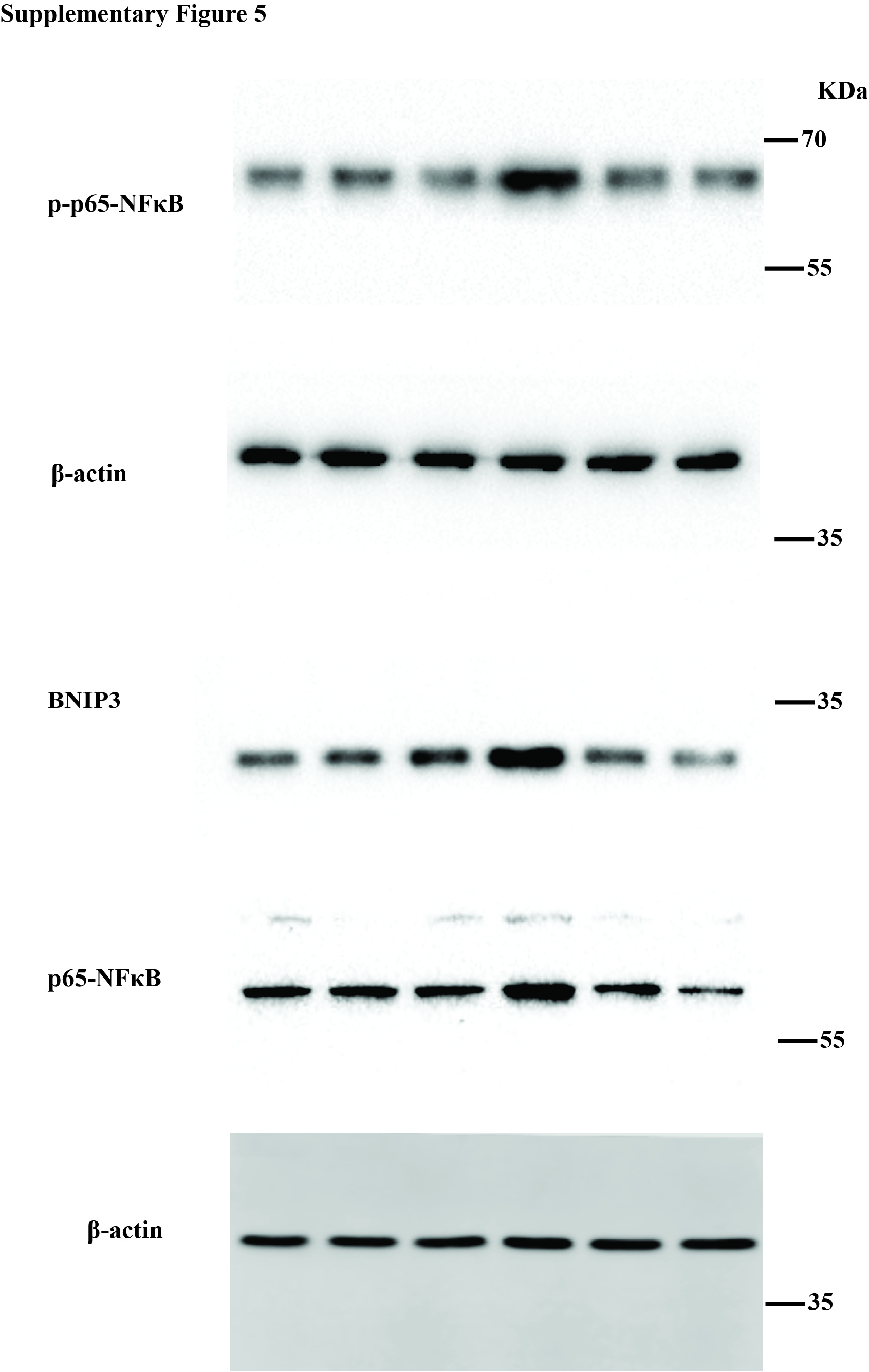

Supplement: FIGURE S5 — Molecular weight markers to gel data and scans in Figure 6A. [file Image_5.JPEG]
